# Bryostatin analog: improving on Nature's design

**DOI:** 10.18632/oncotarget.460

**Published:** 2012-02-29

**Authors:** Dale Boger

**Affiliations:** ^1^ The Scripps Cancer Research Institute, CA, USA

The article of Felsher and Wender entitled “Picolog, a synthetically-available bryostatin analog, inhibits growth of MYC-induced lymphoma *in vivo”* represents a very important key step enroute to the clinical exploration of members of this natural product class [1]. Although bryostatin 1 entered clinical trials, the difficulty in securing sufficient amounts of material from natural sources to ultimately support its clinical use, even with its extraordinary potency, led to their discontinuation in anticipation of synthetic replacements on the horizon [2]. The candidate replacements in this study have come from the Wender laboratory, using cutting-edge synthetic chemistry to prepare well-conceived designed analogs used to explore the structure-function properties and mechanism(s) of action of molecules in the class. In these efforts, simplified and synthetically more accessible candidate drugs have emerged from a >20 year program that express more potent and/or altered selectivity for the identified biological targets of the natural products [3-5]. One of these, the exceptionally potent “picolog”, has now been examined *in vivo* in collaboration with Felsher [6-7], providing efficacious anti-neoplastic activity in a clinically relevant animal model and compelling data is presented that accessible replacements for bryostatin 1 are on the way.

Thus, the study presents the successful results of the first examination of a bryostatin analog in an animal model indicating that it is both efficacious and well tolerated. The work utilized a conditional model of aggressive, MYC-induced T cell lymphoma relevant to clinical applications of bryostatin 1 and provides a valuable pharmacological tool for studying such oncogene induced malignancies going forward. Additionally, the studies showed that picolog, like bryostatin 1, induces apoptosis and operates by a mechanism involving PKC activation. Here, the authors utilized a novel nano-immuno assay (NIA) that allowed them to precisely quantify markers of PKC pathway activation *in vivo* in real time [8-9]. They found that two distinct phosphorylated forms of MEK2 in the PKC pathway were induced by picolog treatment. With these advances, it will be exciting to see how a series of bryostatin analogs performs in such *in vivo* models and how this correlates with novel *in vivo* biomarkers of their anticipated mechanisms of action.

Although the present study has focused on oncology, bryostatin has also emerged as an exciting lead for treatment of Alzheimer's disease, and for purging latent reservoir stores of HIV that are challenging to access in the treatment of AIDS. As such, the present demonstration of the well tolerated in vivo activity of a simplified bryostatin indicates that they, or the appropriate analog subset, may be especially useful in the treatment of other human diseases going forward. At the very least, they will help identify new mechanisms of action relevant to these diseases and define the pharmacological characteristics candidate drugs should embody.

Beyond this, the work is especially significant on several levels. First, by combining the power of state-of-the-art synthetic chemistry, fundamental chemical and structural design principles, modern biology and pharmacology, and innovative technology, new candidate drugs have been created that will not only likely find their way into the clinic, but have or will define new targets and mechanisms of action by which human disease may be treated. Second, and more subtle, the work going forward will undoubtedly define which subset of the constellation of biological properties of bryostatin are responsible for the productive activity useful for treating human disease and identify which bryostatin structural features convey or potentiate these properties. These latter studies provide opportunities to improve on Nature's design, providing drugs that are not only more accessible, but are also more tailored for an intended human use.
